# Disruption of clathrin-dependent trafficking results in the failure of grass carp reovirus cellular entry

**DOI:** 10.1186/s12985-016-0485-7

**Published:** 2016-02-16

**Authors:** Hao Wang, Weisha Liu, Fei Yu, Liqun Lu

**Affiliations:** National Pathogen Collection Center for Aquatic Animals, Shanghai Ocean university, Shanghai, 201306 China; Key Laboratory of Aquatic Genetic Resources of Ministry of Aquaculture, Shanghai Ocean University, Shanghai, 201306 China

**Keywords:** *Ctenopharyngodon idellus*, Clathrin, GCRV, Ammonium chloride, Viral entry

## Abstract

**Background:**

Grass carp reovirus (GCRV) is responsible for viral hemorrhagic disease in cultured grass carp (*Ctenopharyngon idellus*). GCRV is a non-enveloped, double-stranded RNA virus in the genus *Aquareovirus*, of the family *Reoviridae*, which encodes seven structural proteins (VP1-VP7) and five nonstructural proteins (NS80, NS38, NS31, NS26, and NS16). To date, the mechanism of GCRV entry into CIK *Ctenopharyngon idellus* kidney (CIK) cells remains poorly understood.

**Results:**

Here, we present a study of the GCRV internalization mechanism in CIK cells. Our results indicated that GCRV infection was inhibited by chlorpromazine, the specific inhibitor for clathrin-mediated endocytosis. Colocalization of GCRV virions with endogenous clathrin was observed during early infection by confocal microscopy. Moreover, GCRV infection of CIK cells depended on acidification of the endosome. This was indicated by significant inhibition of viral infection following prophylactic treatment with the lysosomotropic drugs chloroquine or ammonium chloride. In addition, the disturbance of dynamin activity blocked GCRV entry, which confirmed the dynamin-dependent nature of clathrin-mediated endocytosis.

**Conclusion:**

Our findings suggest that GCRV might enter CIK cells via clathrin-mediated endocytosis in a pH-dependent manner. Additionally, dynamin is critical for efficient viral entry.

**Electronic supplementary material:**

The online version of this article (doi:10.1186/s12985-016-0485-7) contains supplementary material, which is available to authorized users.

## Background

Over the last three decades, the aquaculture industry has rapidly become the most efficient producer of healthy food in the world and China is one of the primary sources of aquaculture production [1]. According to official data from 2008, the total area devoted to aquaculture reached 5.63 million hectares in China [2]. Production of grass carp (*Ctenopharyngodon idellus*) constitutes the largest aquaculture industry of finfish in China. However, a hemorrhagic disease that is frequently reported in grass carp has severely affected the production of this form of aquaculture [3]. Hemorrhagic disease, caused by the grass carp reovirus (GCRV), is one of the major diseases of grass carp in China [4]. GCRV is a member of the genus *Aquareovirus*, family *Reoviridae. Orthoreovirus* and *Aquareovirus* constitute two genera in the *Reoviridae* family of double-stranded RNA viruses [5]. Seven *Aquareovirus* species (*Aquareovirus* A-G) have been recognized by the International Committee for the Taxonomy of Viruses (ICTV) [6]. In addition, GCRV has been recognized as the most pathogenic of the isolated aquareoviruses reported to date. GCRV also serves as a relevant model system for understanding the diversity and conservation of this large family of dsRNA viruses.

The GCRV virion has an icosahedral capsid with a diameter of 60–70 nm, composed of two protein shells and no envelope [7]. The genome of GCRV contains 11 segments of linear double-stranded RNA [4, 8]. The eleven genome segments encode seven structural proteins (VP1 to VP7) and five nonstructural proteins [7]. Functional information for the GCRV-encoded proteins is extrapolated from the knowledge of their homolog proteins encoded by mammalian reoviruses. The VP1 - VP4 and VP6 proteins are the components of the viral core, and the outer GCRV capsid is composed of 200 trimers of VP5 - VP7 heterodimers. Three copies of the fingerlike VP7 are stacked on top of three VP5 copies and form a VP5 - VP7 complex [9]. Five nonstructural proteins (NS80, NS38, NS31, NS26, and NS16) may also play a role in the viral replication cycle [10]. Indeed, Shao et al. reported that NS80 was essential for the formation of the viral cytoplasmic inclusion structure, resulting in the recruitment of NS38 and the minor core protein, VP4 to these inclusions [11].

The process of a viral infection includes cell binding, penetration, and delivery of the viral genome into a permissive cell [12]. Enveloped viruses enter cells via a membrane fusion reaction driven by conformational changes of specific viral envelope proteins [13]. This membrane fusion is mediated by viral envelope glycoproteins that act as membrane fusion proteins [14]. For example, the fusogenic activity of some envelope glycoproteins, such as the influenza virus hemagglutinin (HA), is activated by a low pH that initiates these conformational changes [15]. Since nonenveloped viruses lack a lipid bilayer, viral entry is not dependent on membrane fusion [16, 17]. Most viruses take advantage of endocytic pathways which allow them to cross membrane barriers and deliver the genome into either the cytosol or nucleus for replication. Endocytosis is involved in sampling the extracellular milieu and also serves to regulate various processes initiated at the cell surface [18]. Multiple forms of host endocytosis have been utilized by viruses, including clathrin-mediated [18, 19], caveolar-mediated [20, 21], micropinocytosis [22], as well as the less well-characterized clathrin and caveolae-independent endocytosis pathways [23]. Mammalian reoviruses (MRV) utilize multiple endocytic pathways for cell entry [24], however, little is known regarding the mechanism of aquareovirus, particularly GCRV entry. In this study, we used cultured *Ctenopharyngon idellus* kidney (CIK) cells to investigate the mechanism of GCRV entry by confocal scanning optical microscopy and an endocytosis inhibition assay.

## Methods

### Cells and viruses

The CIK cell line [25], was derived from grass carp was maintained in M199 (GibcoBRL) media supplemented with 10 % fetal calf serum (Biosource, USA), 50 U of penicillin/mL, and 50 mg/mL streptomycin. The cells were incubated at 28 °C without additional CO_2_. The viral strain, GCRV-JX01 is a laboratory stock. The viral stocks were prepared by passage in CIK cells and purification as previously described [26]. Differential centrifugation was used to extract the GCRV particles from the collected culture supernatant: CIK cell fragments were removed at 8,500 g for 30 min at 4 °C and then the GCRV particles were concentrated at 80,000 g for 3 h at 4 °C. All sample grids were examined under a transmission electron microscope (Hitachi 7000-FA) as previously described [27].

### Chemicals

Ammonium chloride (NH_4_Cl), chloroquine (CQ), nystatin, Filipin III, and dimethyl sulfoxide (DMSO) were purchased from Sigma. Chlorpromazine (CPZ), dynasore and LY294002 were purchased from Selleck. CPZ, NH_4_Cl, and CQ were dissolved in water while the rest of drugs were dissolved in dimethyl sulfoxide (DMSO). NH_4_Cl and CQ affect endocytic pH levels by accumulating in protonated forms within the acidic compartments of cells, effectively binding H^+^ ions. Dynasore acts as a potent inhibitor of endocytic pathways that depend on dynamin by rapidly blocking coated vesicle formation within seconds of administration [28]. The antibiotic nystatin is a sterol binding agent and acts to remove membrane cholesterol. This is essential for both the maintenance of caveolae and for the ability of caveolae to detach from the plasma membrane [29]. Chlorpromazine is a cationic amphiphilic drug that causes clathrin lattices to assemble on endosomal membranes simultaneously to prevent coated pit assembly at the cell surface by controlling AP-2 membrane-binding [30]. Caveolae disappears in cells that are depleted of cholesterol and the exposure of cells to sterol binding agents (e.g., filipin III) provides a useful tool for reducing the caveolae density in the endothelium [31]. PMA-stimulated macropinocytosis can be inhibited by the PI3K inhibitor LY294002, which blocks PI3K functions downstream of PLCγ1 and DAG [31]. A Muse Count and Viability Kit was purchased from Millipore (The Muse^TM^ Count and Viability Kit, Millipore). Cell Counting Kit 8 was purchased from Dojindo (CCK-8; Dojindo Molecular Technology, Gaithersburg, MD). All other reagents, were purchased from Thermo Fisher Scientific (Bremen) and Sigma.

### Confocal imaging

The fluorescence images were obtained using an inverted confocal microscope with appropriate barrier and excitation filters optimized for FITC visualization (Olympus® FluoView FV1000). Signals were captured by a digital camera imaging system (DP30/IX71 CCD, Olympus). The optimal conditions were defined to be the reaction in which maximum fluorescent signals were observed with no background.

### Cytotoxicity assay

Cell cytotoxicity was estimated by measuring both the cell metabolic activity (MTT assay) and the membrane integrity test (The Muse^TM^ Count and Viability Kit, Millipore). Cells were seeded in 96-well plates at a density of 2 × 10^6^ cells/well, grown for 12 h and then treated with inhibitors at the indicated concentrations for 1 h at 4 °C. After two washes with M199 medium, cell viability was determined using CCK-8 (Dojindo Molecular Technologies, USA) according to the manufacturer’s instructions. Untreated cells were used as the controls. After adding the CCK-8 solution, absorbance at 450 nm was measured using a plate-reading luminometer (GloMax®-Multi+ Detection System). Cells were grown in a 24-well plate format and treated with inhibitors at the indicated concentrations for 1 h at 4 °C, before assaying with a Muse Cell Analyzer (Merck Millipore, USA). Floating and adherent cells were collected after the various treatments. Cells were washed twice with PBS and resuspended in PBS for a cell viability assay. The cell viability was ascertained with the Muse Count and Viability Assay Kit (Merck Millipore, USA). The calculations were performed automatically, and the viability profiles (dot plots) were displayed using the Muse™ Count and Viability Software Module. The experiment was carried out in triplicate, and the error bars represent the standard deviation.

### Western blot

For a Western blotting analysis, cells were lysed in the wells with lysis buffer (Thermo Scientific) containing a protease inhibitor cocktail. Protein concentrations were determined with a Bio-Rad protein assay kit. Exactly 100 μg of each sample was boiled in SDS-PAGE loading buffer (Beyotime Institute of Biotechnology). Samples were separated by electrophoresis on a 10 % polyacrylamide gel and transferred to 0.45 μm Immuno-Blot Polyvinylidene fluoride (PVDF) membrane (Merck Millipore, Darmstadt, Germany). After blocking in 5 % (wt/vol) skim milk at room temperature for 1 h, membranes were incubated with a homemade polyclonal antibody against VP5 of GCRV-JX01 at a dilution of 1:1,000, GAPDH at a dilution of 1:3000(Santa Cruz Biotechnology) or Clathrin heavy chain at a dilution of 1:2000 in PBS-T buffer with 5 % skim milk at 4 °C overnight. Horseradish peroxidase (HRP)-conjugated Goat Anti-Rabbit IgG (Santa Cruz Biotechnology) or HRP affinipure anti-mouse IgG(H+L) (EarthOx Biotech) was applied at a dilution of 1:3,000 in PBS-T for 1 h at 37 °C. The signal was developed by ECL Plus Western blot analysis kit (Amersham Pharmacia Biotech, Taiwan, China).

### Effect of inhibitors on GCRV-JX01 infection

Cells were grown in a 24-well plate format and pretreated with inhibitors or DMSO (condition equal to the volume of dynasore used for the 50 μM) at indicated concentrations for 1 h at 4 °C then infected with GCRV-JX01 at an MOI of 5. After 1 h of adsorption at 0 °C, cultures were quickly warmed to 28 °C to start the infection and incubated for another 30 min to allow viral internalization. Non-internalized viruses were then removed by three washes with M199 medium [12]. After treatment, the cells were incubated at 28 °C with various inhibitors as described above. All inhibitors were present throughout all the experiment. For GCRV-JX01 infection analysis, cells were grown until 12 h post-infection (hpi), and the supernatant was collected and assayed for the level of infectious virus by a standard TCID_50_ assay [32]. To ensure adequate statistical analysis of GCRV infection, cells were plated in triplicate for each treatment. The expression of GCRV-VP5 protein at 12 hpi was also detected by Western blot.

### Colocalization analysis of internalized GCRV with clathrin

CIK Cells grown on glass coverslips were incubated with GCRV virions (MOI = 50) on ice for 30 min, and then transferred to 28 °C and incubated for 1 h. Non-internalization GCRV were removed by washing with PBS for three times. The cellular localization of proteins was analyzed by immunofluorescent assay. After fixing cells for 15 min with paraformaldehyde (4 %), the cells were incubated with anti-GCRV-JX01(1:500) and anti-Clathrin-HC(1:200) antibody, subsequently stained with FITC-labeled anti-mouse antiserum(1:500), Rhodamine-labeled anti-rabbit antiserum(1:500) to get GCRV-FITC (green) and Clathrin-Rhodamine (red), respectively.

### Effect of the inhibitors on GCRV-JX01 entry

Cells were plated onto CultureSlides (BD, Falcon) for 12 h. Then cells were pretreated with 20 mM ammonium chloride (NH_4_Cl) in water, 50 μM dynasore in DMSO or DMSO for 1 h at 4 °C. Cells were then adsorbed with GCRV-JX01 virions at an MOI of 20. After 1 h of viral adsorption at 0 °C, warm medium with inhibitors described above was quickly added. All inhibitors were present throughout all the experiment. At 30 min post-infection (mpi), non-internalized viruses were removed and washed three times with PBS. Then cells were fixed with 4 % paraformaldehyde and permeabilized with 0.1 % Triton X-100 for 10 min at room temperature. Viral particles were detected with mouse antiserum raised against GCRV-JX01-VP5 and counterstained with FITC-conjugated anti-mouse antibodies (Sigma). Coverslips were mounted using Vectashield hard Set with DAPI (Thermo Fisher Scientific). The staining patterns of the cells were examined by confocal microscopy as previously described [24].

### Statistical analysis

Error bars indicate the standard errors of the means. To measure the effect of the inhibitors on GCRV-JX01 infection, mean values for a minimum of triplicate samples were compared using paired (normalized) Student’s *t*-tests. A value of *p* < 0.05 was considered to be statistically significant using GraphPad statistics software.

## Results

### Cytotoxicity of pharmaceutical inhibitors

**Fig. 1:**
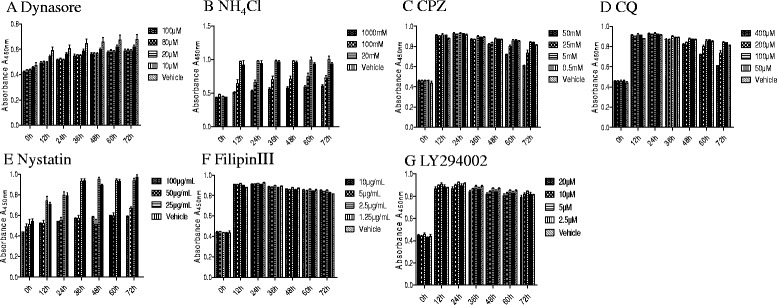
The viability of cells after administration of various inhibitors. MTT assay was performed to monitor the cell viability. *P < 0.05; **P < 0.01; ***P < 0.001. Values were shown as mean ± SD

To further identify the pathway(s) required for GCRV infection, various inhibitors were used to study endocytosis and their effects on viral entry were investigated. Firstly, the cytotoxic effects of these drugs on CIK cells were determined. Cell proliferation and viability were measured using a CCK-8 and Muse Viability Assay Kit according to the manufacturer’s protocol. As shown in Fig. 1 and Additional file 1: Figure S1, the concentrations of inhibitors that had little cytotoxic effect on CIK cells were chosen for the subsequent experiments.

### GCRV infection is clathrin-dependent

**Fig. 2:**
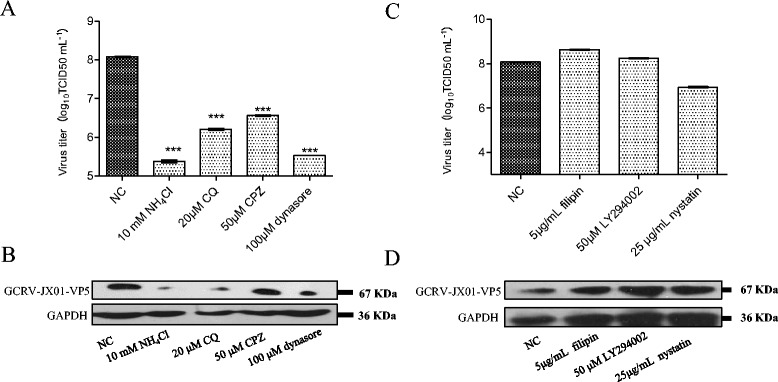
**Effects of inhibitors on GCRV infection. a** Virus yield in CIK cells pretreated with vehicle, 10 mM NH_4_Cl, 20 μM CQ, 50 μM CPZ, or 100 μM of dynasore. **b** Western blotting analysis to monitor viral replication level. Cells were treated with various inhibitors as described above. After 12 h of incubation, cells were lysed and processed for Western blot analysis of GCRV-VP5 protein expression with polyclonal mouse serum. GAPDH was included as an internal loading control for the Western blots. NC denotes the negative control treated with vehicle. **c** Virus yield in CIK cells pretreated with vehicle, 5 μg/mL Filipin III, 50 μM LY294002, or 25 μg/mL nystatin. **d** Western blotting analysis to monitor the viral replication level. Cells were treated with inhibitors as described above. After 12 h of incubation, cells were lysed and processed for Western blot analysis of GCRV-VP5 protein expression with polyclonal mouse serum. GAPDH was included as an internal loading control for the Western blots. NC denotes the negative control treated with vehicle. Each experimental point is the sum of triplicate experiments with two titer assays. Error bars represent the standard errors of the mean. Asterisks represent a significant difference from the control (un-paired *t*-test)

To explore the early stages of GCRV infection in CIK cells, inhibitors that specifically block different viral entry pathways were used. NH_4_Cl, CQ, and CPZ were used to inhibit clathrin-mediated endocytosis [33]. Firstly, we examined the ability of CPZ to block viral infection. CPZ causes clathrin lattices to assemble on endosomal membranes while inducing the disassembly of clathrin-coated pits at the plasma membrane [33, 34]. As shown in Fig. 2a, in the CIK cells treated with CPZ, viral infection was inhibited at 12 hpi. Moreover, a productive infection of GCRV was monitored by Western blot to demonstrate the expression level of GCRV-VP5 (Fig. 2b). To verify the effects of CPZ on clathrin-mediated endocytosis, we examined the effect of NH_4_Cl and CQ on viral titers. As expected, a reduction was found in the level of GCRV infectivity following NH_4_Cl or CQ treatment. A reduction of 3 log units at 10 mM NH_4_Cl and 2 log units at 20 μM CQ was observed (Fig. 2a). Likewise, the expression levels of the viral protein, GCRV-VP5, also diminished after NH_4_Cl or CQ infection (Fig. 2b). The caveolae-dependent and macropinocytosis pathways were considered to be the most likely candidates for GCRV entry. LY294002 is an inhibitor of PI3K by preventing cup closure but not ruffle closure. PI3-kinase is also implicated in several other viral entry pathways. For example, the endocytic entry of adenovirus [35], and human herpesvirus 8 (HHV-8) [36] has been proposed to require PI3-kinase activity based on evidence that PI3-kinase inhibitors, such as LY294002, block viral entry. Furthermore, disruption of caveolae by filipin III and nystatin could provide a useful tool for separating intracellular transport mechanisms. In particular, this will distinguish between entry mediated by coated and noncoated vesicles, as well as transvascular transport in the endothelium occurring via paracellular or transcellular pathways. The effects of filipin III, LY294002, and nystatin on GCRV infection were determined by TCID_50_ and Western blot. As shown in Fig. 2c and d, the treatment of CIK cells with 5 μg/mL filipin III, 50 μM LY294002, or 25 μg/mL nystatin did not impair GCRV infection.

**Fig. 3:**
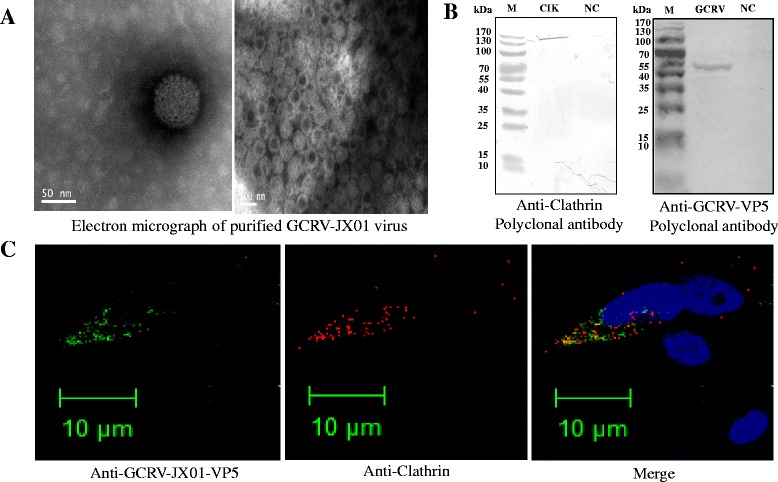
**Colocalization of GCRV-JX01 and clathrin in endosomes. a** Purified intact GCRV virions. Magnification, ×30,000 (panels A), Scale bars represent 50 nm and 100 nm. **b** Western blot analysis of purified GCRV particles and clathrin in CIK cells with polyclonal antibody against VP5 or anti-Clathrin-heavy chain antibody. **c** CIK cells were incubated with GCRV-JX01 virions (MOI = 50) on ice for 30 min, and then transferred to 28 °C. Cells were washed, fixed and analyzed by IFA with anti-GCRV JX01 VP5 antibody(green) plus anti-Clathrin-heavy chain antibody(red). Scale bars represent, 10 μM

If GCRV enters cells through clathrin-mediated endocytosis, GCRV virons and clathrin should colocalize in endosomes during early infection. Since clathrin-HC is conserved from yeast to mammals [37], we used a mammalian anti-clathrin-HC antibody to detect grass carp clathrin. To prove our hypothesis, GCRV virion was purified by ultracentrifugation (Fig. 3a). The presence of outer capsid protein VP5 in the purified virion and clathrin in CIK cells was confirmed by Western blotting analysis with anti-VP5 and anti-clathrin polyantibody, respectively (Fig. 3b). As shown in Fig. 3c, an overlay of the red (Clathrin) and green (GCRV virions) channels indicated that colocalized GCRV virions and endogenous clathrin could be observed in CIK cells (Fig. 3c, yellow-orange color in merged panel).

Therefore, these results collectively indicate that GCRV utilizes a clathrin-dependent pathway for cellular entry.

### GCRV entry is dynamin-dependent

**Fig. 4:**
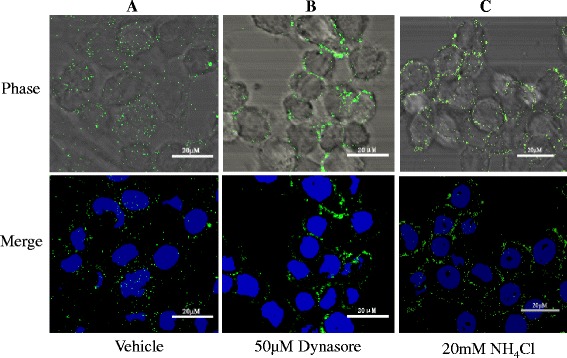
**Effects of dynasore and NH**_**4**_ **Cl on GCRV entry**. GCRV-JX01 virions at an MOI of 20 were adsorbed to CIK cells that had been pretreated with (**a**) DMSO(condition equal to the volume of dynasore used for the 50 μM), (**b**) 50 μM dynasore in DMSO or (**c**) 20 mM ammonium chloride (NH_4_Cl) in water. After 1 h of viral adsorption at 0 °C, warm medium with inhibitors described above was quickly added. At 30 min post-infection (mpi), non-internalized viruses were removed and washed three times with PBS. Then cells were fixed with 4 % paraformaldehyde and permeabilized with 0.1 % Triton X-100 for 10 min at room temperature. The samples were labeled with an anti-GCRV VP5 polyantibody (**green**) and DAPI (**Blue**). Scale bars represent 20 μM

**Fig. 5:**
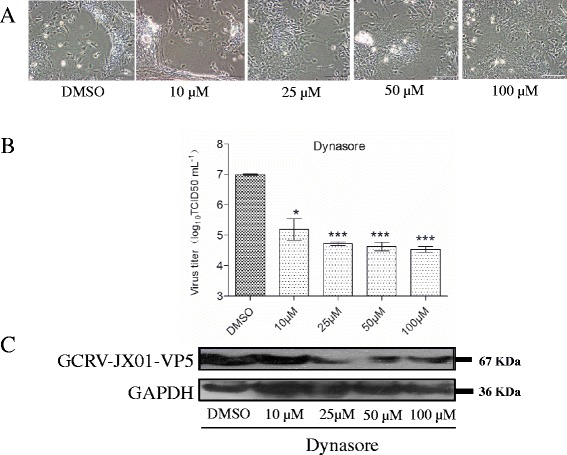
**Effect of dynasore on the production of progeny virions**. Cells were incubated with dynasore at the indicated concentrations. **a** CPE of GCRV infection viewed under a visible light phase microscope. **b** TCID_50_ assay of viral yield in harvested supernatants. Each experimental point is the sum of triplicate experiments with two titer assays. Error bars represent the standard errors of the mean. Asterisks represent a significant difference from the control (unpaired *t-*test). **c** Western blot analysis to monitor viral replication level. Cells were treated with dynasore as described above. After 12 h of incubation, cells were lysed and processed for a Western blot analysis of GCRV-VP5 protein expression with polyclonal mouse serum. GAPDH was included as an internal loading control for the Western blots. NC denotes the negative control treated with the vehicle

Dynamin is a large GTPase of approximately 100 kDa. It mediates several forms of endocytosis, as well as vesicle formation from various intracellular organelles through its ability to tubulate and sever membranes [19]. To determine if dynamin is essential for a productive infection, we assessed the growth of GCRV in the presence of dynasore. As shown in Figures, viral entry (Fig. 4b) and propagation (Fig. 5) were both inhibited by dynasore. Figure 5a shows the CPE in CIK cells pretreated with various doses of dynasore at 12 hpi, and the suppression of viral replication in infected cells was quantitatively analyzed by a TCID_50_ assay. We found that DMSO treated CIK cells showed a typical CPE profile, including extensive cell fusion and necrosis, while treatment with dynasore resulted in protection of cells from viral infection (Fig. 5). Notably, the treatment of CIK cells with 25 mM resulted in a 2 log inhibition of viral replication, compared with the vehicle (DMSO) treatment. As determined by Western blot, the expression of GCRV-VP5 in GCRV-infected cells was significantly inhibited by 50 μM of dynasore. These data indicate that GCRV takes advantage of a dynamin-dependent endocytic pathway to infect CIK cells.

### GCRV entry is dependent on a low pH environment

**Fig. 6:**
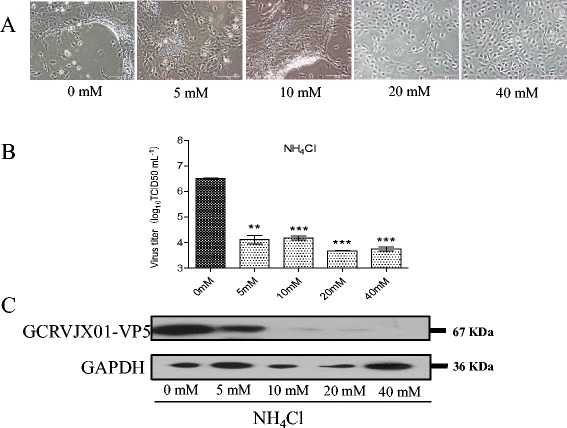
**Effect of NH**_**4**_ **Cl on the production of progeny virus.** Cells were incubated with NH_4_Cl at the indicated concentrations. **a** CPE of GCRV infection viewed under a visible light phase microscope. **b** TCID_50_ assay of virus yield in the supernatants. Each experimental point is the sum of triplicate experiments with two titer confirmation assays. Error bars represent standard errors of the mean. Asterisks represent a significant difference from the control (unpaired *t*-test). **c** Western blot analysis. Cells were treated with NH_4_Cl as described above. After 12 h of incubation, cells were lysed and processed for Western blot analysis of GCRV-VP5 protein expression with polyclonal mouse serum. GAPDH was included as an internal loading control for the Western blots. NC denotes the negative control treated with the vehicle

To explore whether GCRV entry is pH-dependent, we infected CIK cells with GCRV in the presence of the weakly basic amines NH_4_Cl or CQ, which selectively raise the pH of the endosome and lysosome. This raises the pH of the endosomes and inhibits pH-dependent viral membrane fusion. These effects have been well-studied against Human Immunodeficiency Virus [38] and Sindbis Virus [39] replication, and are currently in clinical trials. Figure 2a indicates that GCRV infection was inhibited in CIK cells treated with either 10 mM NH_4_Cl or 20 μM CQ. Specifically, we observed a decrease of 3 log units and 2 log units, respectively compared to the control. The relative infection of GCRV following each treatment was determined by Western blot (Fig. 2b). NH_4_Cl was used at different concentrations to assess the inhibitory effect on GCRV internalization and infection because NH_4_Cl is less toxic to cells than the other agents (Additional file 1: Figure S1). We used confocal microscopy to visualize GCRV particle uptake in CIK cells that had been pretreated with either the DMSO (vehicle) or 20 mM NH_4_Cl and then synchronously infected with GCRV particles. While DMSO-treated cells exhibited a significant uptake of GCRV particles into the cytoplasm at 30 min post-infection, we found that the viral particles were concentrated at the periphery of the NH_4_Cl treated cells (Fig. 4c). We studied the growth of GCRV in CIK cells covered with medium containing various concentrations (0 mM, 5 mM, 10 mM, 20 mM, and 40 mM) of NH_4_Cl. Figure 6a shows the CPE in CIK cells treated with various doses of NH_4_Cl at 12 hpi and the suppression of viral replication in infected supernatants was quantitatively analyzed using a TCID_50_ assay. Virally-induced typical CPE was observed at 12 hpi with the vehicle and GCRV (Fig. 6a). As shown in Fig. 6b, the viral replication level in the CIK cells pretreated with NH_4_Cl was lower than that of the control group. This indicated that NH_4_Cl could inhibit GCRV entry. Moreover, the expression of the GCRV major capsid protein VP5 was impaired at a concentration of 10 mM NH_4_Cl and was almost undetectable at 40 mM NH_4_Cl (Fig. 6c). These results indicate that the entry of GCRV is dependent on a low pH.

## Discussion

Entry is the initial step in the viral infection cycle and is necessary for understanding tissue tropism and pathogenicity. To propagate, both enveloped and nonenveloped viruses must deliver their genome and accessory proteins into host cells, across either the endosome or plasma membrane [17]. To achieve this, various host endocytoic pathways have been utilized by viruses, including clathrin-mediated endocytosis, caveolae, macropinocytosis, as well as other nonclathrin and noncaveolae routes. Cell entry of MRV virions has been reported to use both dynamin-dependent and dynamin-independent endocytic pathways [24]. Although MRV and GCRV share a physical resemblance with double-layered icosahedral capsids, GCRV differs from MRV in some respects, including the absence of the hemagglutinin spike, sigma-1 (σ1) protein, and a disproportionate number of dsRNA genomic segments [7]. Recently, endocytic research on mammalian cells, (e.g., the genus *Orthoreovirus* and murine rotavirus [MRV]) has been well-studied, while little progress has been made on the genus *Aquareovirus*, particularly GCRV.

In this study, the entry mechanism of GCRV was investigated in detail by applying endocytosis inhibition assays. We used various specific inhibitors to block different entry pathways to investigate the early stages of GCRV infection in CIK cells. In the colocalization experiment, the fact that GCRV virions colocalized with clathrin indicated that GCRV virions should utilize clathrin-dependent pathway of endocytosis for cell entry.

The large GTPase dynamin was reported to be essential for clathrin-coated vesicle formation and transportation [40]. The ability of dynamin to regulate actin remodeling and to associate with membrane-bending proteins could provide an additional driving force to expand pores and permit the release of the virion core. Dynamin plays a significant role in many forms of endocytosis because vesicle scission at the plasma membrane is required for subsequent internalization [19]. Dynasore inhibits the GTPase activity of dynamin1, dynamin2, and Drp1 (the mitochondrial dynamin) but not of other small GTPases. Dynasore acts as an inhibitor of endocytic pathways known to depend on dynamin by rapidly blocking coated vesicle formation within seconds of administration [28]. In this study, we used dynasore to determine whether dynamin was involved in GCRV entry. Our data (Figs. 4b and 5) showed that GCRV entry and replication was dependent on dynamin.

Clathrin-mediated endocytosis also provides endocytic vesicles as an acidified environment for viruses that require a low pH during the first stages of infection to initiate capsid destabilization and genome uncoating [34]. Here, we demonstrate that a productive GCRV infection is pH-dependent, implying that the penetration of GCRV may occur in the endosome or lysosome and a low pH is required for the uncoating process. Clathrin-mediated endocytosis has been clearly demonstrated by CPZ treatment for viruses, such as Vesicular Stomatitis Virus [41], Japanese Encephalitis Virus (JEV) [29], Hepatitis C Virus [42] and Human Polyomavirus JC Virus [43]. We used CPZ to inhibit clathrin-dependent endocytosis and nystatin and filipin III to inhibit caveola-dependent endocytosis. We found that GCRV infection was significantly inhibited by CPZ while virion entry was not suppressed by nystatin and filipin III. The possible involvement of macropinocytosis in GCRV infection was also examined in this study.

We found that cells treated with LY294002 exhibited a minimal inhibition of GCRV infection after 12 h. Clathrin-mediated endocytosis is a continuous process, and for viral entry, it is rapid and efficient [44]. In contrast, the internalization processes of virus endocytosis via caveolae are slower than entry by endocytosis [45]. Our laboratory [26] and others have demonstrated that CIK cells produce a typical cytopathic effect (CPE) of forming large syncytia at 12 h post-infection at a low multiplicity of infection (MOI) [26]. Additionally, subviral particles without outer layer proteins could be seen in the cytoplasm as early as 4 hpi [46]. Our data combined with previous evidence suggests that GCRV virions are rapidly endocytosed by a constitutive process.

## Conclusions

In summary, the evidence presented here indicates that GCRV entry into CIK cells is predominantly via a pH-dependent, clathrin-mediated endocytic pathway and is dependent on dynamin. Further studies are required to identify specific host receptor(s) that are involvement in GCRV cellular entry.

## Additional file

Additional file 1: Figure S1. Cell viability was assessed using a Muse Count and Viability Kit to determine the safety of applied inhibitors. (PPT 917 kb)

## Abbreviations

dsRNA: double-stranded RNA
GCRV: grass carp reovirus
hpi: hour post infection
mpi: minute post infection
MRV: mammalian orthoreovirus

## References

[1] Wang Q, Cheng L, Liu J, Li Z, Xie S, De Silva SS. Freshwater aquaculture in PR China: trends and prospects. Rev Aquacult. 2015;7(4):283–302.

[2] Xie B, Qin J, Yang H, Wang X, Wang Y-H, Li T-Y (2013). Organic aquaculture in China: A review from a global perspective. Aquaculture.

[3] Jiang Y. Hemorrhagic disease of grass carp: status of outbreaks, diagnosis, surveillance, and research. Bamidgeh. 2009;61:188–197.

[4] Qiu T, Lu R-H, Zhang J, Zhu Z-Y (2001). Complete nucleotide sequence of the S10 genome segment of grass carp reovirus (GCRV). Dis Aquat Organ.

[5] Kim J, Tao Y, Reinisch KM, Harrison SC, Nibert ML (2004). Orthoreovirus and Aquareovirus core proteins: conserved enzymatic surfaces, but not protein–protein interfaces. Virus Res.

[6] Mohd Jaafar F, Goodwin AE, Belhouchet M, Merry G, Fang Q, Cantaloube J-F (2008). Complete characterisation of the American grass carp reovirus genome (genus Aquareovirus: family Reoviridae) reveals an evolutionary link between aquareoviruses and coltiviruses. Virology.

[7] Attoui H, Fang Q, Jaafar FM, Cantaloube J-F, Biagini P, de Micco P (2002). Common evolutionary origin of aquareoviruses and orthoreoviruses revealed by genome characterization of Golden shiner reovirus, Grass carp reovirus, Striped bass reovirus and golden ide reovirus (genus Aquareovirus, family Reoviridae). J Gen Virol.

[8] Zhang Q, Gui J-F (2015). Virus genomes and virus-host interactions in aquaculture animals. Sci China Life Sci.

[9] Cheng L, Fang Q, Shah S, Atanasov IC, Zhou ZH (2008). Subnanometer-resolution structures of the grass carp reovirus core and virion. J Mol Biol.

[10] Fan C, Shao L, Fang Q (2010). Characterization of the nonstructural protein NS80 of grass carp reovirus. Arch Virol.

[11] Shao L, Guo H, Yan L-M, Liu H, Fang Q (2013). Aquareovirus NS80 recruits viral proteins to its inclusions, and its C-terminal domain is the primary driving force for viral inclusion formation. PLoS One.

[12] Huang J, Li F, Wu J, Yang F (2015). White spot syndrome virus enters crayfish hematopoietic tissue cells via clathrin-mediated endocytosis. Virology.

[13] Bressanelli S, Stiasny K, Allison SL, Stura EA, Duquerroy S, Lescar J (2004). Structure of a flavivirus envelope glycoprotein in its low‐pH‐induced membrane fusion conformation. EMBO J.

[14] Han X, Bushweller JH, Cafiso DS, Tamm LK (2001). Membrane structure and fusion-triggering conformational change of the fusion domain from influenza hemagglutinin. Nat Struct Mol Biol.

[15] Skehel JJ, Wiley DC (2000). Receptor binding and membrane fusion in virus entry: the influenza hemagglutinin. Annu Rev Biochem.

[16] Corcoran JA, Duncan R (2004). Reptilian reovirus utilizes a small type III protein with an external myristylated amino terminus to mediate cell-cell fusion. J Virol.

[17] Smith AE, Helenius A (2004). How viruses enter animal cells. Science.

[18] Kumari S, Swetha M, Mayor S (2010). Endocytosis unplugged: multiple ways to enter the cell. Cell Res.

[19] Mayor S, Pagano RE (2007). Pathways of clathrin-independent endocytosis. Nat Rev Mol Cell Biol.

[20] van den Berg LM, Ribeiro CM, Zijlstra-Willems EM, de Witte L, Fluitsma D, Tigchelaar W (2014). Caveolin-1 mediated uptake via langerin restricts HIV-1 infection in human Langerhans cells. Retrovirology.

[21] Zhang Y, Whittaker GR (2014). Influenza entry pathways in polarized MDCK cells. Biochem Biophys Res Commun.

[22] Rossman JS, Leser GP, Lamb RA (2012). Filamentous influenza virus enters cells via macropinocytosis. J Virol.

[23] Quirin K, Eschli B, Scheu I, Poort L, Kartenbeck J, Helenius A (2008). Lymphocytic choriomeningitis virus uses a novel endocytic pathway for infectious entry via late endosomes. Virology.

[24] Schulz WL, Haj AK, Schiff LA (2012). Reovirus uses multiple endocytic pathways for cell entry. J Virol.

[25] Zuo W, Qian H, Xu Y, Du S, Yang X (1986). A cell line derived from the kidney of grass carp. J Fish China.

[26] Wang T, Li J, Lu L (2013). Quantitative in vivo and in vitro characterization of co-infection by two genetically distant grass carp reoviruses. J Gen Virol.

[27] Fang Q, Seng E, Ding Q, Zhang L (2008). Characterization of infectious particles of grass carp reovirus by treatment with proteases. Arch Virol.

[28] Macia E, Ehrlich M, Massol R, Boucrot E, Brunner C, Kirchhausen T (2006). Dynasore, a cell-permeable inhibitor of dynamin. Dev Cell.

[29] Nawa M, Takasaki T, Yamada K-I, Kurane I, Akatsuka T (2003). Interference in Japanese encephalitis virus infection of Vero cells by a cationic amphiphilic drug, chlorpromazine. J Gen Virol.

[30] Wang L-H, Rothberg KG, Anderson R (1993). Mis-assembly of clathrin lattices on endosomes reveals a regulatory switch for coated pit formation. J Cell Biol.

[31] Schnitzer JE, Oh P, Pinney E, Allard J (1994). Filipin-sensitive caveolae-mediated transport in endothelium: reduced transcytosis, scavenger endocytosis, and capillary permeability of select macromolecules. J Cell Biol.

[32] He Y, Xu H, Yang Q, Xu D, Lu L (2011). The use of an in vitro microneutralization assay to evaluate the potential of recombinant VP5 protein as an antigen for vaccinating against Grass carp reovirus. Virol J.

[33] Mercer J, Schelhaas M, Helenius A (2010). Virus entry by endocytosis. Annu Rev Biochem.

[34] Hernaez B, Alonso C (2010). Dynamin-and clathrin-dependent endocytosis in African swine fever virus entry. J Virol.

[35] Pickles RJ, McCarty D, Matsui H, Hart PJ, Randell SH, Boucher RC (1998). Limited entry of adenovirus vectors into well-differentiated airway epithelium is responsible for inefficient gene transfer. J Virol.

[36] Pati S, Cavrois M, Guo H-G, Foulke JS, Kim J, Feldman RA (2001). Activation of NF-κB by the human herpesvirus 8 chemokine receptor ORF74: evidence for a paracrine model of Kaposi’s sarcoma pathogenesis. J Virol.

[37] Kirchhausen T (2000). Clathrin. Annu Rev Biochem.

[38] Savarino A, Boelaert JR, Cassone A, Majori G, Cauda R (2003). Effects of chloroquine on viral infections: an old drug against today’s diseases. Lancet Infect Dis.

[39] Cassell S, Edwards J, Brown DT (1984). Effects of lysosomotropic weak bases on infection of BHK-21 cells by Sindbis virus. J Virol.

[40] Sever S, Damke H, Schmid SL (2000). Dynamin: GTP controls the formation of constricted coated pits, the rate limiting step in clathrin-mediated endocytosis. J Cell Biol.

[41] Sun X, Yau VK, Briggs BJ, Whittaker GR (2005). Role of clathrin-mediated endocytosis during vesicular stomatitis virus entry into host cells. Virology.

[42] Blanchard E, Belouzard S, Goueslain L, Wakita T, Dubuisson J, Wychowski C (2006). Hepatitis C virus entry depends on clathrin-mediated endocytosis. J Virol.

[43] Pho M, Ashok A, Atwood WJ (2000). JC virus enters human glial cells by clathrin-dependent receptor-mediated endocytosis. J Virol.

[44] Marsh M, Helenius A (1989). Virus entry into animal cells. Adv Virus Res.

[45] Beer C, Andersen DS, Rojek A, Pedersen L (2005). Caveola-dependent endocytic entry of amphotropic murine leukemia virus. J Virol.

[46] Zou G, Fang Q (1999). Study on replication and morphogenesis of the Grass Carp Reovirus (GCRV) in CIK cells. Virol Sin.

