# Supplementary material for: Disruption of clathrin-dependent trafficking results in the failure of grass carp reovirus cellular entry
Source: Virol J. 2016 Feb 16;13:25. doi: 10.1186/s12985-016-0485-7 (PMC4754963; doi:10.1186/s12985-016-0485-7)
Supplement: Additional file 1: Figure S1. — Cell viability was assessed using a Muse Count and Viability Kit to determine the safety of applied inhibitors. (PPT 917 kb) [file 12985_2016_485_MOESM1_ESM.ppt]

## Slide 1
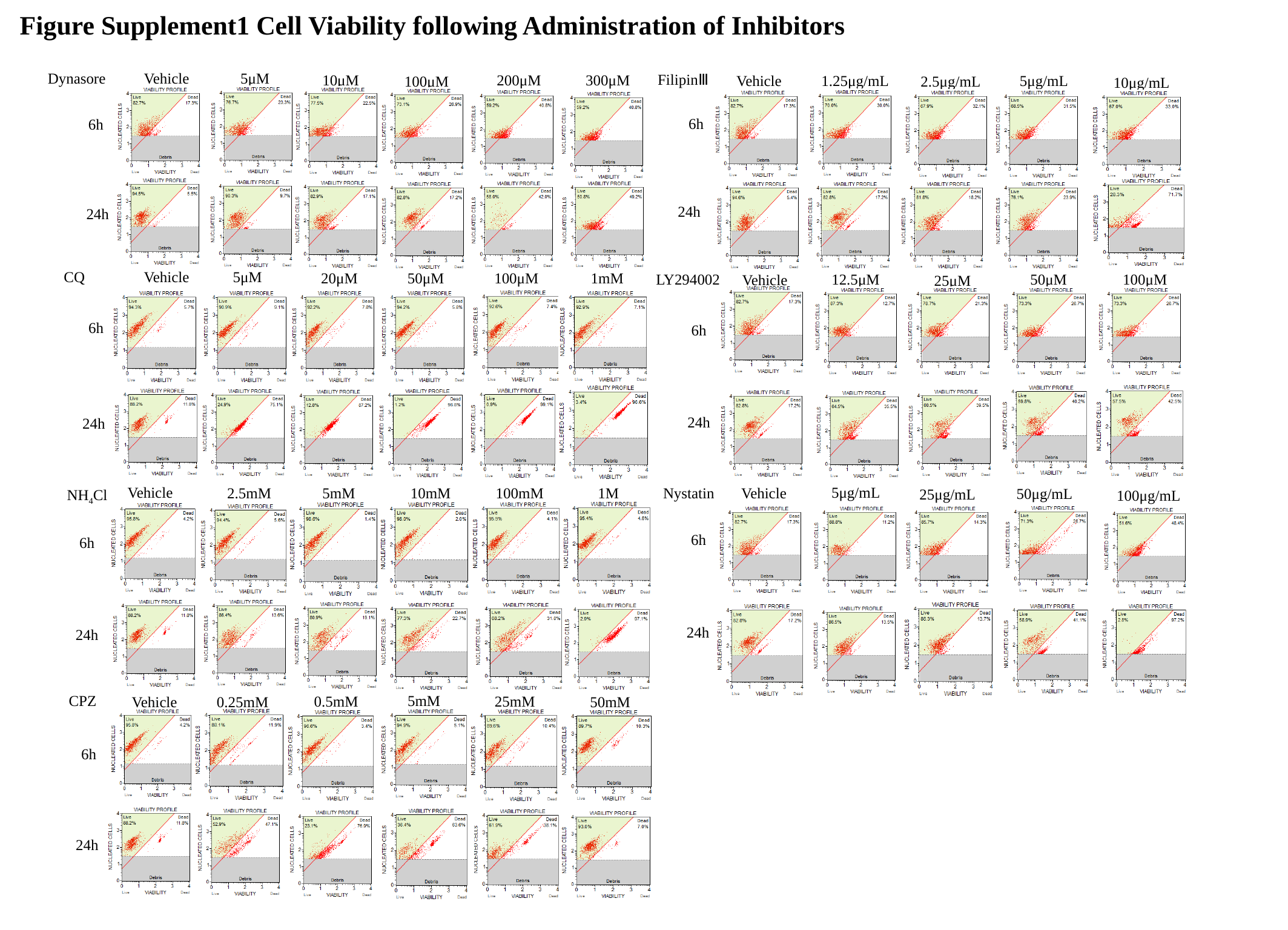

Figure Supplement1 Cell Viability following Administration of Inhibitors
Dynasore
Vehicle
 5μM
FilipinⅢ
 10μM
300μM
 200μM
Vehicle
 1.25μg/mL
 5μg/mL
 100μM
 2.5μg/mL
 10μg/mL
 6h
6h
 24h
24h
Vehicle
CQ
5μM
50μM
100μM
1mM
20μM
 100μM
 50μM
LY294002
 12.5μM
 Vehicle
 25μM
6h
6h
24h
24h
 Vehicle
 5μg/mL
Nystatin
 Vehicle
2.5mM
5mM
10mM
100mM
1M
 50μg/mL
 25μg/mL
NH4Cl
100μg/mL
6h
6h
24h
24h
CPZ
5mM
25mM
0.5mM
Vehicle
0.25mM
50mM
6h
24h
